# Nephrectomy Induces Severe Bone Loss in Mice Expressing Constitutively Active TGFβ Receptor Type I

**DOI:** 10.3390/ijms26062704

**Published:** 2025-03-17

**Authors:** Parichart Toejing, Ohnmar Myint, Asada Leelahavanichkul, Somyoth Sridurongrit, Matthew B. Greenblatt, Sutada Lotinun

**Affiliations:** 1 Center of Excellence in Skeletal Disorders and Enzyme Reaction Mechanism, Department of Physiology, Faculty of Dentistry, Chulalongkorn University, Bangkok 10330, Thailand; lookplanoi_@hotmail.com (P.T.); ohnmarmyint.rl@gmail.com (O.M.); 2 Division of Immunology, Department of Microbiology, Faculty of Medicine, Chulalongkorn University, Bangkok 10330, Thailand; aleelahavanit@gmail.com; 3 Department of Anatomy, Faculty of Science, Mahidol University, Bangkok 10400, Thailand; somyoth.sri@mahidol.ac.th; 4 Department of Pathology and Laboratory Medicine, Weill Cornell Medicine, New York, NY 10065, USA; mag3003@med.cornell.edu; 5 Skeletal Health and Orthopedic Research Program, Hospital for Special Surgery, New York, NY 10065, USA

**Keywords:** TGFβ, osteoblast, osteoclast, bone, chronic kidney disease

## Abstract

Transforming growth factor beta (TGF-β), a master regulator of renal fibrosis, is the hallmark of chronic kidney disease (CKD) progression, and CKD worsens bone remodeling. However, the effects of the dysregulation of *TGF-β* signaling on bone remodeling during CKD have not been investigated. Here, we determined the effects of TGF-β receptor I (*TβRI*) overexpression under the control of Mx1-Cre on bone remodeling in CKD mice (*Mx1;TβRI^CA^*-CKD mice). Our results demonstrated that kidney fibrosis and serum urea nitrogen levels were elevated in *Mx1;TβRI^CA^*-CKD mice compared to WT-CKD, indicating that *TβRI* overexpression exacerbated renal injury during CKD. Serum calcium was decreased, while PTH was enhanced, in *Mx1;TβRI^CA^*-CKD mice. *Mx1;TβRI^CA^*-CKD mice displayed severe osteopenia as assessed by uCT in both femurs and mandibles. An histomorphometric analysis showed that tibial cancellous bone volume was decreased in *Mx1;TβRI^CA^*-CKD. Likewise, mRNA expression levels of an osteoclastogenesis marker, *Tnfsf11/Tnfrsf11b,* was increased, and osteoblast marker genes *Runx2* and *Sp7* were decreased in *Mx1;TβRI^CA^*-CKD mice. *Mx1;TβRI^CA^*-CKD mice displayed increased inflammatory cytokines levels. Together, our results indicated that in the setting of CKD, *TβRI* overexpression induced both CKD progression and the dysregulation of bone remodeling, leading to severe bone loss. As such, these data provide an avenue for the future development of therapeutics for CKD-induced osteoporosis.

## 1. Introduction

Transforming growth factor beta (TGF-β) superfamily signaling has fundamental roles in bone homeostasis. Three TGF-β isoforms, TGF-β1, β2 and β3, are found in mammalian tissue. These three isoforms are expressed in bone; however the TGF-β1 isoform is the most abundant. TGF-β act through binding to receptor complexes that are comprised of type I and type II receptors at the cell surface, leading to them initiating intracellular signaling through two signaling pathways, the Smad and Smad-independent pathways. After the receptors are activated, the phosphorylation of Smad2/3 induces the formation of a complex with Smad4, subsequently translocated into a nucleus for regulating the transcription of target genes. Non-Smad signaling includes mitogen-activated protein kinase (MAPK) pathways, including ERK, JNK, p38 MAPK and IKK, PI3K/Akt and Rho family GTPases [1]. Numerous studies have reported that the dysregulation of TGF-β signaling leads to diverse impacts on bone remodeling. Mice lacking TGF-β1 die during embryonic development or develop severe inflammatory disorders and die within 3–4 weeks after birth [2]. TGF-β1 deletion reduced osteoblast apoptosis via the p44/42 MAPK pathway in the mouse osteoblast cell line MC3T3-E1 [3]. Expressing active TGF–β1 in osteoblastic cells stimulated osteoarthritis. In addition, the knockout of *Tgfbr2* in nestin-positive skeletal progenitors reduced the development of osteoarthritis in an anterior cruciate ligament transection (ACLT) osteoarthritis mouse model [4]. Furthermore, TGF-β1 increased osteoclast differentiation through a Smad2/3 cascade mediated by a TRAF6–TAB1–TAK1 complex signaling pathway in bone marrow cells [5]. Matsunobu et al. [6] demonstrated that conditional knockout TβRI mice using Dermo1-cre in skeletal progenitor cells showed short and wide long bones and reduced trabecular bones. *Prx1Cre;Tgfbr2^f/f^* mice had short limbs and defects in joint development [7]. Mice lacking *Tgfbr2* showed a decreased proliferation and maturation of immature osteoblasts, as well as bone volume [8]. In addition, we previously demonstrated that transgenic mice that overexpress *TβRI* were osteopenic due to increases in osteoclast numbers and decreases in osteoblast numbers [9].

Chronic kidney disease (CKD) causes a dysregulation of the parathyroid hormone (PTH), fibroblast growth factor 23 (FGF23) and vitamin D homeostasis, which induces abnormalities of calcium and phosphate metabolism. The interrupted mineral metabolism from CKD impairs bone homeostasis, which is known as CKD–mineral bone disease (CKD-MBD) [10]. Increased bone turnover and PTH levels, together with deceases in cortical thickness, were found in adenine-induced CKD male and female C57Bl/6 mice [11]. A CKD rat model induced by a two-step 5/6 nephrectomy impairs bone regeneration in calvaria or femurs [12], and cortical mandibular bone thickness was reduced in CKD mice [13]. Moreover, TGF-β has been generally considered as a key mediator in the pathogenesis of renal fibrosis. Increased TGF-β1 plasma levels, mesangial expansion and extracellular matrix (ECM) protein accumulation, which result in the development of glomerulosclerosis and tubulointerstitial fibrosis, were found in transgenic mice overexpressing *TGF-β1* [14]. A transgenic mouse model that overexpressed *TGF-β1* in the renal tubules displayed fibrosis [15]. Mechanistically, TGF-β1 can induce renal fibrosis via both Smad and Smad-independent signaling pathways, leading to myofibroblast activation, the excessive production of ECM and the inhibition of ECM degradation. Mice lacking Smad3 (*Smad3^ex8/ex8^*) are protected against renal fibrosis following ureteral obstructive nephropathy [16]. An enhancement in Smad3 signaling and renal fibrosis were observed in mice with *Smad2* conditional knockouts [17]. The deletion of Smad4 suppressed the binding of Smad3 to the COL1A2 promoter, thus inhibiting the fibrotic response and renal fibrosis [18]. In addition, TGF-β1 induced the expression of type I collagen via MKK3-p38 signaling that caused the development of fibrotic lesions [19]. Moreover, high glucose, advanced glycation end products (AGEs), inflammation and angiotensin II also induce renal fibrosis by stimulating TGF-β production [17]. Anti-TGF-β treatments and Smad3 inhibitor, SIS3, inhibit renal fibrosis in STZ-induced diabetic nephropathy in *Tie2-cre;Loxp-EGFP* mice [20]. The administration of anti-TGF-βl suppresses the production of extracellular matrix in acute mesangial proliferative glomerulonephritis [21]. Mice and human primary renal interstitial cells treated with miR-1908 decreased renal fibrosis via the suppressed protein expression of TGF-β1, Smad2/3 and MMP-2 [22]. In this study, we investigated the effects of TGF-β receptor I (TβRI) overexpression on bone remodeling during CKD; we generated CKD by a 5/6 nephrectomy with angiotensin II in transgenic mice expressing constitutively active *TβRI* under the control of Mx1-Cre (*Mx1;TβRI^CA^* mice). We hypothesized that *TβRI* overexpression might be an important driving factor for renal injury, subsequently contributing to the dysregulation of bone remodeling, leading to more severe bone loss. Our results demonstrated that serum urea nitrogen, kidney fibrosis and PTH were elevated in *Mx1;TβRI^CA^*-CKD mice when compared to CKD or *Mx1;TβRI^CA^* mice. μCT analysis showed that *Mx1;TβRI^CA^*-CKD mice had a decreased cortical thickness and cancellous bone volume in both femurs and mandibles. Histomorphometric analysis showed a significant decrease in cancellous bone volume, together with increased osteoclast numbers and decreased osteoblast numbers. Similarly, mRNA expression levels of *Tnfsf11/Tnfrsf11b*, a major regulator of osteoclastogenesis, were increased, and osteoblast marker genes *Runx2* and *Sp7* were decreased in *Mx1;TβRI^CA^*-CKD mice. Inflammatory cytokine TNF-α, IL-6 and IL-23 levels were elevated in *Mx1-TβRI^CA^*-CKD mice. These data indicated that *TβRI* overexpression caused more renal injury during CKD, which increased the dysregulation of mineral homeostasis and inflammation, leading to dramatically increased bone loss.

## 2. Results

### 2.1. CKD Mice Display Characteristics of Abnormal Renal Function, and More Severe Characteristics Were Found in Mx1;TβRI^CA^ Mice

**Figure 1:**
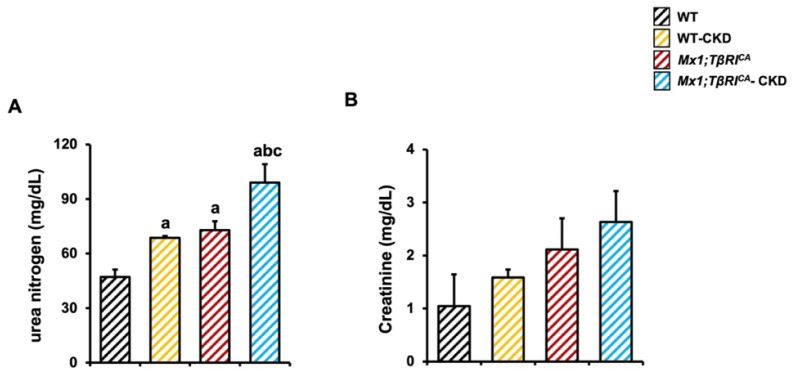

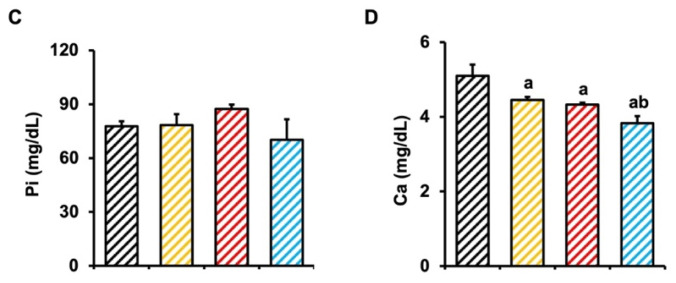
Serum chemistries in experimental groups. Serum levels of (**A**) urea nitrogen (*n* = 5–6), (**B**) creatinine (*n* = 4), (**C**) phosphorus (*n* = 5–7) and (**D**) calcium (*n* = 4–5). Data are mean ± SEM. ^a^ *p* < 0.05 compared to WT, ^b^ *p* < 0.05 compared to WT-CKD, and ^c^ *p* < 0.05 compared to *Mx1;TβRI^CA^*.

To assess renal function, serum levels of urea nitrogen and creatinine were investigated. Compared to WT, WT-CKD or *Mx1;TβRI^CA^* mice, serum urea nitrogen levels were enhanced in *Mx1;TβRI^CA^*-CKD mice, indicating that a 5/6 nephrectomy with ANG II treated-mice was an effective approach to induce CKD (Figure 1A). Serum creatinine levels showed a tendency towards an increase but did not significantly differ between experimental groups (Figure 1B). These data suggested that the overexpression of *TβRI* during CKD induced more severe renal injury, as seen in the *Mx1;TβRI^CA^*-CKD mice.

As the kidneys are an important regulator of mineral homeostasis, we next determined phosphorus and calcium levels. There was no significant different in serum phosphorus levels among the experimental groups (Figure 1C). However, serum levels of calcium significantly decreased in WT-CKD, *Mx1;TβRI^CA^* and *Mx1;TβRI^CA^*-CKD compared to the WT control. In addition, serum calcium levels in *Mx1;TβRI^CA^*-CKD were significantly reduced when compared to WT-CKD (Figure 1D). Together, these results indicated that the disturbance in renal function during CKD affected mineral homeostasis, and this disturbance was more severe in *Mx1;TβRI^CA^* mice.

### 2.2. TβRI Induces Renal Fibrosis During CKD

**Figure 2:**
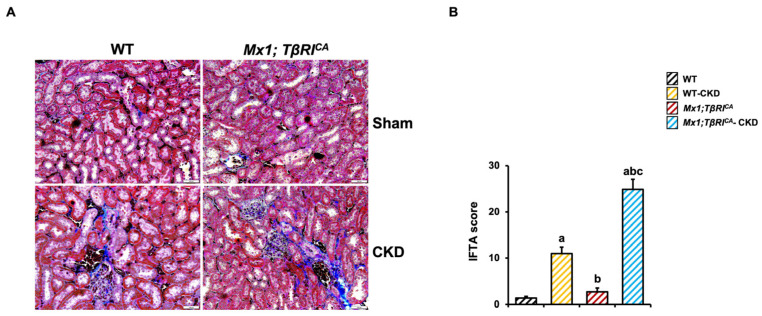
*Mx1;TβRI^CA^*-CKD mice show induced renal fibrosis. (**A**) Masson’s trichrome staining of kidneys. Blue color indicates fibrosis with accumulation of collagen. Scale bar = 100 μm. (**B**) IFTA score in kidneys (*n* = 5). Results are mean ± SEM. ^a^ *p* < 0.05 compared to WT, ^b^ *p* < 0.05 compared to WT-CKD, and ^c^ *p* < 0.05 compared to *Mx1;TβRI^CA^*.

Next, we confirmed the decline in kidney function by measuring kidney fibrosis. The renal interstitial fibrosis and tubular atrophy (IFTA) score showed a significant increase in WT-CKD and Mx1;TβRI^CA^-CKD mice compared to WT controls (Figure 2B). Interestingly, Mx1;TβRI^CA^-CKD mice showed a significantly enhanced IFTA score compared to WT-CKD mice. A two-way ANOVA indicated an interaction between the overexpression of TβRI and CKD. This result indicated that TβRI overexpression induced renal fibrosis during CKD.

### 2.3. Mx1;TβRI^CA^-CKD Mice Have Increased Serum PTH Levels

**Figure 3:**
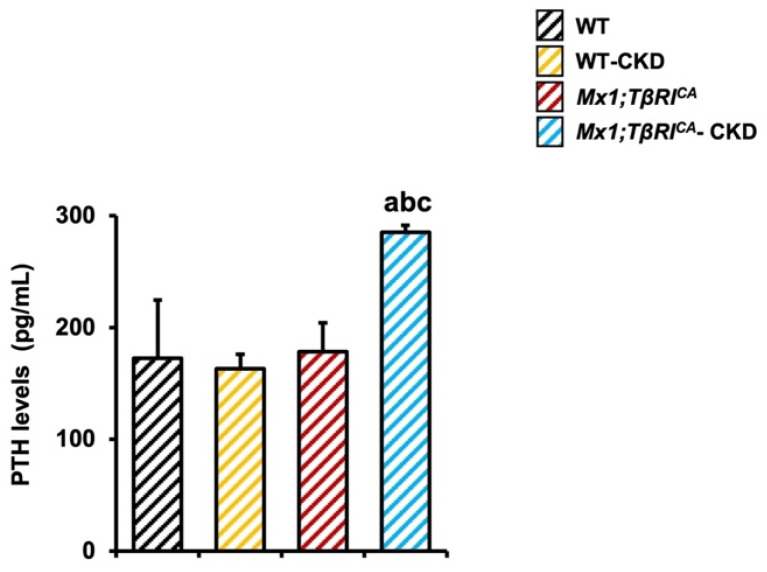
Serum PTH is increased in *Mx1;TβRI^CA^*-CKD mice. Data are mean ± SEM. ^a^ *p* < 0.05 compared to WT, ^b^ *p* < 0.05 compared to WT-CKD, and ^c^ *p* < 0.05 compared to *Mx1;TβRI^CA^* (*n* = 4).

PTH maintains circulating blood calcium levels by stimulating bone resorption. *Mx1;TβRI^CA^*-CKD mice displayed significantly increased serum PTH levels compared to WT, WT-CKD or *Mx1;TβRI^CA^* mice (Figure 3), indicating that the overexpression of *TβRI* induced PTH secretion during CKD.

### 2.4. Mx1;TβRI^CA^-CKD Mice Have Severe Cancellous and Cortical Bone Loss in Femurs and Mandibles

**Figure 4:**
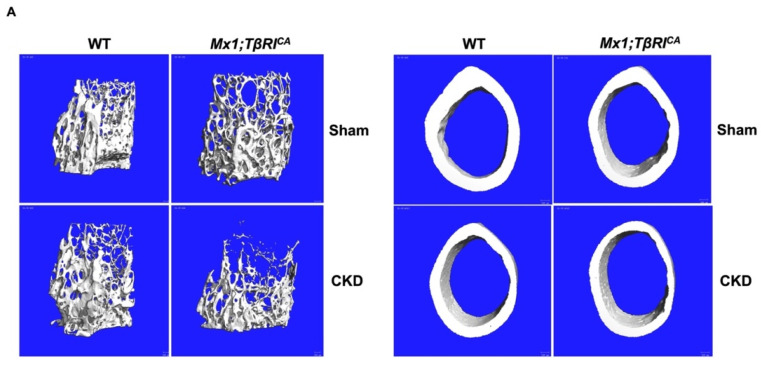

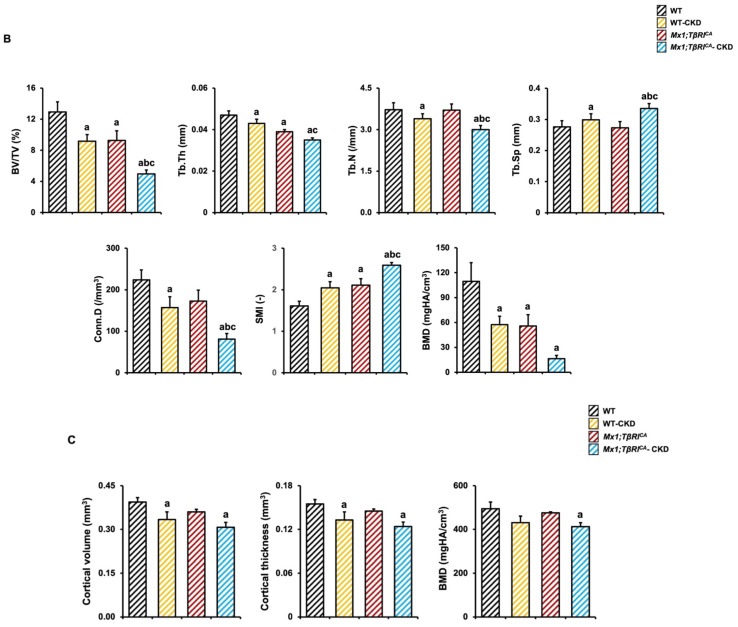
Overexpression of *TβRI* increases femoral bone loss in CKD mice. (**A**) Representative μCT images of cancellous and cortical bone in femurs. (**B**) μCT analysis of femoral cancellous bone (*n* = 4–5). (**C**) μCT analysis of femoral cortical bone (*n* = 4–5). Data are mean ± SEM. ^a^ *p* < 0.05 compared to WT, ^b^ *p* < 0.05 compared to WT-CKD, and ^c^ *p* < 0.05 compared to *Mx1;TβRI^CA^* (*n* = 4–7).

**Figure 5:**
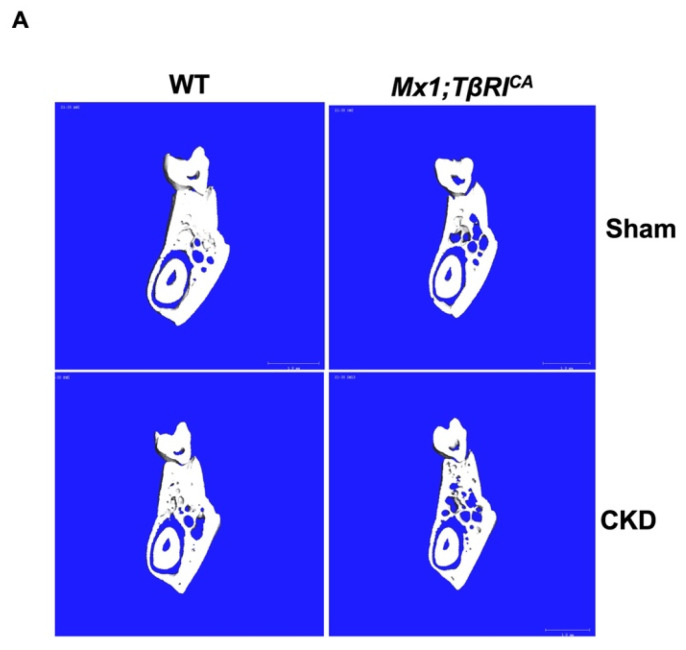

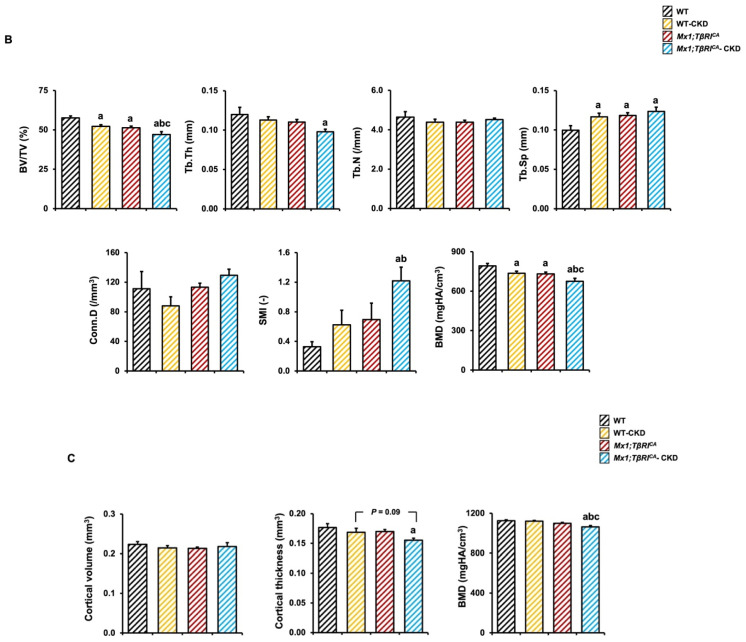
Overexpression of *TβRI* increases mandibular bone loss in CKD mice. (**A**) Representative μCT images of cancellous and cortical bone in mandibles. (**B**) μCT analysis of mandibular cancellous bone (*n* = 5–6). (**C**) μCT analysis of mandibular cortical bone (*n* = 5). Data are mean ± SEM. ^a^ *p* < 0.05 compared to WT, ^b^ *p* < 0.05 compared to WT-CKD, and ^c^ *p* < 0.05 compared to *Mx1;TβRI^CA^* (*n* = 4–7).

To evaluate whether the constitutive activation of *TβRI* affected femoral and mandibular cancellous and cortical bone during CKD, µCT analysis was performed. µCT representative images of femoral and mandibular bone in all groups are shown in Figure 4A and Figure 5A. WT-CKD and *Mx1;TβRI^CA^* mice were osteopenic in both femurs and mandibles. Interestingly, severe osteopenia was observed in transgenic mice overexpressing *TβRI* with CKD. Quantifications of femoral cancellous and cortical parameters are shown in Figure 4B,C. Compared to WT-CKD mice, the femoral cancellous bone volume, trabecular number and connectivity density significantly decreased, while trabecular separation and the structural model index significantly increased in *Mx1;TβRI^CA^*-CKD mice (Figure 4B). Similarly, decreases in cancellous bone volume, trabecular thickness and bone mineral density, together with increases in trabecular separation and the structural model index, were found in the mandibular bone of *Mx1;TβRI^CA^*-CKD mice compared to WT mice (Figure 5B). Cancellous bone volume and bone mineral density were decreased, while the structural model index was significantly increased, in *Mx1;TβRI^CA^*-CKD mice compared to WT-CKD mice. Cortical thickness and bone mineral density were significantly reduced in both the femurs and mandibles of *Mx1;TβRI^CA^*-CKD mice compared to WT mice (Figure 4C and Figure 5C). *Mx1;TβRI^CA^*-CKD mice showed a significantly decreased mandibular bone mineral density compared to WT-CKD (Figure 5C). Therefore, *TβRI* overexpression might be an important driving factor, which causes more severe bone loss during CKD.

### 2.5. Mx1;TβRI^CA^-CKD Mice Have Decreased Osteoblasts and Increased Osteoclasts in Tibiae

**Figure 6:**
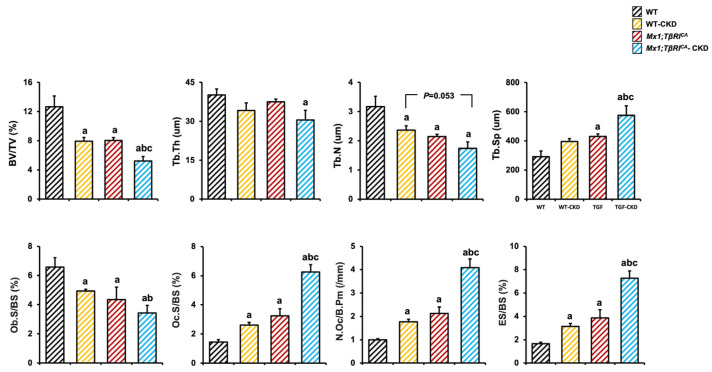
Tibial bone histomorphometric data of experimental groups (*n* = 4–7). Data are mean ± SEM. ^a^ *p* < 0.05 compared to WT, ^b^ *p* < 0.05 compared to WT-CKD, and ^c^ *p* < 0.05 compared to *Mx1;TβRI^CA^* (*n* = 4–7).

A tibial bone histomorphometric analysis revealed a significant decrease in cancellous bone volume, trabecular number and osteoblast surface per bone surface, together with an increase in osteoclast surface per bone surface and osteoclast number per bone perimeter, in WT-CKD mice, *Mx1;TβRI^CA^* mice and *Mx1;TβRI^CA^*-CKD mice compared to WT mice (Figure 6). In addition, in comparison to WT-CKD mice, cancellous bone volume and osteoblast surface per bone surface were significantly reduced, while trabecular separation, osteoclast surface per bone surface, osteoclast number per bone perimeter and eroded surface per bone surface were significantly increased, in *Mx1;TβRI^CA^*-CKD mice. A two-way ANOVA displayed an additive effect between *TβRI* overexpression and CKD in osteoclast surface per bone surface, osteoclast number per bone perimeter and eroded surface per bone surface. These results suggested that during CKD, *TβRI* overexpression exerted an additive effect on decreased bone formation, as seen in a reduced osteoblast surface and increased bone resorption, as seen in increasing of osteoclast surface resulting in severe bone loss.

### 2.6. Mx1;TβRI^CA^ Mice Have Decreased Osteoblast and Increased Osteoclast-Related Gene Expression Following CKD

**Figure 7:**
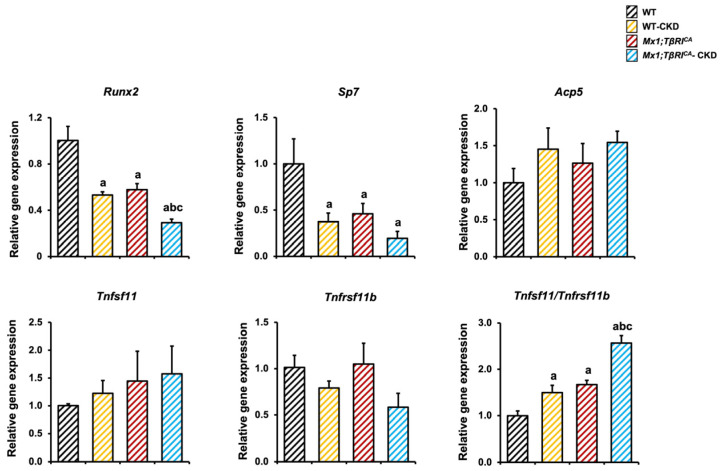
Constitutive *TβRI* activation decreases osteoblast and increases osteoclast-related gene expression in femurs of CKD mice. Data are mean ± SEM. ^a^ *p* < 0.05 compared to WT, ^b^ *p* < 0.05 compared to WT-CKD, and ^c^ *p* < 0.05 compared to *Mx1;TβRI^CA^* (*n* = 3–4).

To study the cellular basis of the alterations in bone formation and bone resorption, a qPCR was performed to determine the expression of osteoblast- and osteoclast-associated transcripts in femurs. *Runx2* and *Sp7*, key transcription factors for osteoblast differentiation, were significantly reduced in WT-CKD, *Mx1;TβRI^CA^* and *Mx1;TβRI^CA^*-CKD compared to WT controls (Figure 7). Interestingly, *Runx2* was significantly decreased in *Mx1;TβRI^CA^*-CKD compared to WT-CKD. There was no significant difference in the expression of *Acp5*, *Tnfsf11* and *Tnfrsf11b*. However, the ratio of *Tnfsf11/Tnfrsf11b*, a major regulator of osteoclastogenesis, was increased in *Mx1;TβRI^CA^*-CKD compared to WT, WT-CKD and *Mx1;TβRI^CA^* mice. These data confirmed that *Mx1;TβRI^CA^*-CKD induced the enhancement of bone loss via decreased osteoblast differentiation and increased osteoclast differentiation at the level of associated changes in gene expression.

### 2.7. Mx1;TβRI^CA^-CKD Mice Have Increased Pro-Inflammatory Cytokines and Decreased Anti-Inflammatory Cytokine Makers Related Bone Loss

**Figure 8:**
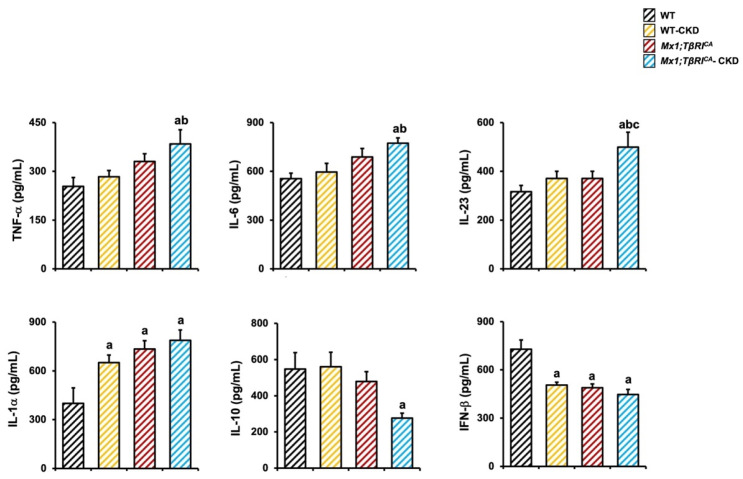
*Mx1;TβRI^CA^*-CKD mice had increased inflammatory cytokines. Serum levels of TNF-α, IL-6, IL-23, IL-1α, IL-10 and IFN-β (*n* = 3–7). Data are mean ± SEM. ^a^ *p* < 0.05 compared to WT, ^b^ *p* < 0.05 compared to WT-CKD, and ^c^ *p* < 0.05 compared to *Mx1;TβRI^CA^*.

As inflammatory cytokines play a role in bone remodeling, we next performed a flow cytometry to measure serum levels of inflammatory mediators. The results showed that TNF-α, IL-6, IL-23 and IL-1α levels significantly increased in *Mx1;TβRI^CA^*-CKD mice compared to WT mice (Figure 8). In addition, *Mx1;TβRI^CA^*-CKD mice showed significantly increased TNF-α, IL-6 and IL-23 levels compared to WT-CKD mice. IL-10 and IFN-β are recognized as anti-inflammatory cytokines. We found that IL-10 levels were decreased in *Mx1;TβRI^CA^*-CKD mice compared to WT mice. The levels of IFN-β were significantly reduced in WT-CKD, *Mx1;TβRI^CA^* and *Mx1;TβRI^CA^*-CKD mice compared to WT mice. An interaction between *TβRI* overexpression and CKD by two-way ANOVA was found in IFN-β levels. This indicated that increases in pro-inflammatory cytokine levels are another factor inducing bone loss in *Mx1;TβRI^CA^*-CKD mice.

## 3. Discussion

Abnormalities in mineral and bone metabolism following chronic kidney disease (CKD) are referred to as CKD–mineral and bone disorder (CKD-MBD) which is associated with an increased risk of bone fractures and other serious complications, ultimately leading to increases in morbidity and mortality. An improved understanding of CKD-MBD is needed. Our previous study reported that the overexpression of *TβRI* induced osteopenia. In the present study, we determined the effects of *TβRI* overexpression on bone remodeling in 5/6 Nx with Ang II administration. Under pathological conditions of CKD, *TβRI* overexpression increased both the progression of renal injury and the dysregulation of bone remodeling, eventually leading to severe bone loss.

5/6 Nx in C57BL/6 mice are resistant to CKD induction. The administration of Ang II 0.75 μg/kg/min was used to overcome the resistance of C57BL/6 mice to CKD [23]. Ang II, the biologically active peptides of the renin–angiotensin system, plays an importance role in regulating renal fibrosis and inflammation. The subcutaneous infusion of Ang II (200 ng/min) in rats caused phenotypic changes in glomerular mesangial cells and desmin in epithelial cells [24]. Moreover, an Ang II 100 ng/min administration for 7 days increased extracellular matrix production and the progression of injury in rat glomerulus [25]. Our results found that serum urea nitrogen levels, a hallmark of renal dysfunction, were increased in *Mx1;TβRI^CA^*-CKD mice compared to WT, WT-CKD and *Mx1;TβRI^CA^* mice, suggesting that *TβRI* overexpression caused severe renal dysfunction. Our results were supported by Zhang et al. [26], who demonstrated an increase in urea nitrogen levels in polycystic kidney disease in a *TGF-β1* overexpression mouse model (*PKD;TGF-β1^CD^* mice). In addition, we also found that renal fibrosis was enhanced in *Mx1;TβRI^CA^*-CKD mice, confirming that *TβRI* overexpression induced renal failure. Previously, 5/6 Nx was found to induce renal fibrosis by enhancing the protein expression of α-SMA, a hallmark of mature myofibroblasts, together with increased collagen I and III, leading to renal dysfunction [27]. Moreover, unilateral ureteral obstruction (UUO)-induced CKD models increased levels of TGF-β and the renal fibrotic genes Col1a1 and Fibronectin-1 [28]. TGF-β has been considered an important mediator in the pathogenesis of renal fibrosis. TGF-β bound its receptor, inducing the phosphorylation of the Smad2/3 complex, and translocated into the nucleus to regulate gene expression, leading to the promotion of fibrosis by increasing collagen I, collagen III and TIMP-1, together with reducing MMP protein expression. In the CKD model, TGF-β1 stimulated the expression of *fibronectin*, *α-SMA*, *collagen I* and *collagen III*, resulting in renal tubulointerstitial fibrosis via the activation of Smad2/3 signaling [29]. Smad3-null (*Smad3^ex8^/^ex8^*) mice blocked EMT and collagen accumulation and also abrogated the monocyte influx that contributed to reduce tubulointerstitial fibrosis following UUO [16].

Serum phosphorus levels were not different among the experimental groups. The progression of CKD reduced the glomerular filtration rate, leading to phosphorus retention. However, to maintain serum phosphorus levels within the normal range, nephrons increased the volume of phosphorus excretion as compensatory mechanisms. Likewise, other evidence found that phosphorus levels are stable or modestly reduced during early CKD [30]. Parathyroid hormone (PTH) is released from the parathyroid gland due to decreases in plasma calcium. PTH not only increases calcium reabsorption by the kidney but also acts on bone to enhance bone resorption. As expected, our results showed that calcium levels were decreased, while PTH levels were enhanced, in *Mx1*-*TβRI^CA^* mice with CKD. Calcium-sensing receptor (CaSR) is a receptor of calcium that is expressed in the parathyroid cell to maintain calcium–PTH homeostasis. During CKD, low calcium levels or calcium deficiency diminished the activation of calcium and CaSR, resulting in increased PTH secretion [31]. Moreover, TGF-β increased the number of functional PTH receptors through increasing both PTH binding and cAMP formation in cultured ROS 17/2.8 osteosarcoma cells [32]. Taken as a whole, our study indicated that the overexpression of *TβRI* enhanced renal dysfunction, as shown by increasing serum urea nitrogen and renal fibrosis, which contributed to the dysregulation of calcium and PTH levels during CKD.

TGF-β and its downstream signaling are important regulators of bone remodeling. The overexpression or null mutations of *TGF-β* pathway signaling mediators affected the balance of bone remodeling [33]. As expected, uCT and histomorphometry analyses found that the overexpression of *TβRI* in *Mx1-TβRI^CA^* mice induced osteopenia. Our previous study reported that *TβRI* overexpression caused increases in osteoclast numbers and decreases in osteoblast numbers which related to the suppression of Hedgehog signaling, leading to reductions in bone mass [9]. In the present study, we confirmed that mRNA expression levels of *Tnfsf11/Tnfrsf1b*, an osteoclastogenesis marker, were increased, together with decreases in the osteoblast marker genes *Runx2* and *Sp7,* in *Mx1-TβRI^CA^* mice. Consistently, *TGF-β*/*Smad3* and class IIa *HDACs* act as corepressors that mediate the repression of *Runx2* function and osteoblast differentiation [34]. *Tnfrsf1b* (OPG) can bind to *Tnfsf11* (RANKL), resulting in the prevention of the interaction of *Tnfsf11* (RANKL) and its receptor, *Tnfrsf11A* (RANK), leading to the suppression of the development and activity of osteoclasts. TGF-β1 induced the expression of *Tnfrsf11A* (RANK), leading to increases in *Tnfrsf11A/Tnfsf11* (RANK/RANKL), which caused NF-κB activation to induce osteoclastogenesis genes’ expression [1]. A reduction in serum OPG and high bone turnover was found in CKD patients with maintenance hemodialysis (HD) therapy [35]. In addition, we found that WT-CKD or *Mx1-TβRI^CA^*-CKD mice were osteopenic compared to WT or *Mx1-TβRI^CA^* mice, respectively. *Mx1-TβRI^CA^*-CKD mice were more severely impacted than WT-CKD mice. The expression levels of *Runx2* were decreased, whereas *Tnfsf11/Tnfrsf1b* was increased in both WT and *Mx1-TβRI^CA^* mice with CKD. Likewise, a previous study reported that *Runx2* expression was decreased in skeletal progenitors from CKD rats compared to normal rats [36]. Collectively, these results suggested that *TβRI* overexpression caused increases in osteoclast numbers and decreases in osteoblast numbers, leading to the dramatic bone loss found during CKD.

Inflammation is generally implied to stimulate bone resorption. We found an enhancement in serum TNF-α, IL-6 and IL-23 levels in *Mx1-TβRI^CA^* -CKD mice compared to WT or WT-CKD mice. TNF-α inhibits *Runx2* expression via the up-regulation of Smurf1, leading to decreased osteoblast differentiation [37]. IL-6 stimulates osteoclast precursors and increased inflammatory mediators [38]. Moreover, the attenuation of trabecular bone loss via the suppression of the IL-6/STAT3 pathway was found in IL-6 gene knockout (IL-6 KO) mice with HFD-induced bone loss [39]. IL-23 stimulated osteoclast precursors, resulting in increased osteoclastogenesis and bone resorption in inflammatory arthritis [40]. In contrast, IL-10 and IFN-β are known as anti-inflammatory cytokines. IL-10 suppresses osteoclast progenitor differentiation into osteoclast precursors [41]. IFN-β inhibited RANKL-induced osteoclastogenesis by decreasing c-Fos [42]. In addition, a previous study reported that TGF-β involved the inflammatory responses. TGF-β1 induced cytokines, such as TNF–α, IL-1β, IL-1 MIP-1α and MMP-1, in cultured fibroblast-like synoviocytes from rheumatoid arthritis (RA) and osteoarthritis (OA) patients [43]. Furthermore, CKD has been shown to increase the degree of systemic inflammation, which is responsible for the progression of bone disorders. During CKD, the levels of adipokines, adhesion proteins such as ICAM-1 and VCAM-1 and uremic toxins were elevated. Moreover, uremic toxins cause increased C-reactive protein (CRP), nitric oxide (NO) and nuclear factor kappa-light-chain-enhancer of activated B cells (NF-κB), resulting in enhanced pro-inflammatory cytokine levels [44]. Together, these results indicated that increases in the expression of osteoclast-associated genes and decreases in osteoblast-associated genes in *Mx1;TβRI^CA^*-CKD mice, resulting in severe bone loss, were mediated by both TGF-β signaling and CKD-related inflammation.

Abnormalities of mineral metabolism due to CKD are recognized as a bone disorder known as CKD-MBD. During CKD progression, the kidney fails to regulate the levels of calcium and phosphorus, leading to increased levels of FGF23 as an attempt to normalize mineral levels. Unfortunately, this process causes decreased 1,25(OH)_2_D_3_ and contributes to enhanced PTH levels, which promote bone resorption by increasing the *Tnfsf11/Tnfrsf1b* ratio. Previous evidence indicated that PTH and TGF-β act to jointly regulate bone resorption and bone formation. After TGF-β binds to TβRII, this leads to phosphorylation of the cytoplasmic domain of PTH1R that integrates PTH signaling to regulate bone remodeling. Moreover, low concentrations of PTH stimulated the mRNA expression of *Runx2* and *Osterix* in the rat osteoblast-like cell line UMR 106 [45]. Numerous lines of evidence have revealed that CKD induces bone loss. CKD mouse caused decreases in cortical mandibular bone thickness [13]. A μCT analysis showed decreased bone strength in the cortical and trabecular bone in an adenine-induced CKD rat model [46]. Herein, we provided the first evidence that the overexpression of *TβRI* increased the severity of renal injury during CKD, which contributed to a dysfunction in mineral hometostasis and subsequent inflammation. These effects increased osteoclasts and decreased osteoblast numbers, leading to enhanced bone loss.

In summary, our findings strongly indicated that the overexpression of *TβRI* exerted additive effects to exacerbate the pathogenesis of CKD and contributed to osteopenia. However, the ability of TGF-β inhibition to rescue these effects will be tested rigorously in future studies.

## 4. Materials and Methods

### 4.1. Animals

*TβRI^CA^* mice with a C57BL/6 background from Dr. Laurent Bartholin were obtained from the Department of Anatomy, Faculty of Science, Mahidol University, Bangkok, Thailand. The animal protocol was approved by the Institutional Animal Care and Use Committee (IACUC) at the Faculty of Medicine, Chulalongkorn University (protocol no. 006/2566) in accordance with the Animal Research: Reporting In Vivo Experiments guidelines (ARRIVE, Singapore). Mice were housed at the Faculty of Medicine, Chulalongkorn University, under a controlled temperature (25 ± 2 °C) and with a 12 h light/dark cycle, standard rodent chow (C.P. Mice Feed, Perfect Companion Group Co., Ltd., Bangkok, Thailand) and water ad libitum.

### 4.2. Generation of Mice Overexpressing TβRI in the Setting of CKD

*TβRI^CA^* mice were crossed with mice expressing Cre recombinase under the control of the interferon-inducible Mx1 promoter to generate *Mx1;TβRI^CA^* mice, according to our previous study [9]. Female mice were used in this study, because constitutively active *TβRI* was knocked into the X chromosome-linked hypoxanthine phosphoribosyl transferase (HPRT) locus to generate *TβRI^CA^* mice. Seven-week-old female mice were classified into four groups, WT, WT-CKD, *Mx1;TβRI^CA^* and *Mx1;TβRI^CA^*-CKD mice. For CKD, a 5/6 nephrectomy model was performed using a two-step surgical procedure under isoflurane anesthesia. Upper and lower poles of the left kidney were cut through a flank incision. A microfibrillar collagen hemostat (Avitene, Davol, Cranston, RI, USA) was used to stop bleeding. After one week, step two was performed by removing the right kidney. Mice were infused with angiotensin II (Val5-Ang II 0.75 mg/kg/min, Sigma-Aldrich, St Louis, MO, USA) dissolved in NSS by a subcutaneous osmotic minipump (Alzet model 1004, Cupertino, CA, USA) for 4 weeks. The osmotic minipump was inserted under isoflurane anesthesia at day 3 after step two 5/6 Nx [23]. Mice were administered an analgesia, tramadol (25 mg/kg), every 12 h for 3 days. At the end of the experiment, the mice were anesthetized with isoflurane and sacrificed by cervical dislocation. Blood samples were collected and kept at −80 °C to measure the serum chemistry and PTH. Left kidneys were kept in 10% neutral buffered formalin for histological analysis. Left femurs, mandibles and tibias were fixed in 70% alcohol for μCT and histomorphometry analysis. Right femurs were kept at −80 °C for qPCR analysis.

### 4.3. μCT

High-resolution images of the femurs and mandibles were acquired using a desktop μCT35, (Scanco Medical, Wangen-Brüttisellen, Switzerland) according to the recommended guidelines [47]. The scanning parameters were subjected to Gaussian filtration and segmentation using a voltage of 70 kV, 113 μA and 7 and 12 μm voxel size for femurs and mandibles, respectively. The threshold was set at 190 and 350 for 300 transverse slices of cancellous and 86 slices of cortical bone for the femurs, respectively. Mandibles were quantified from 200 slices, with the threshold set at 300 and 280 for cancellous and cortical bone. Bone volume (BV/TV, %), trabecular number (Tb.N,/mm), trabecular thickness (Tb.Th, mm), trabecular separation (Tb.Sp, mm), connectivity density (Conn.D,/mm^3^), structural model index (SMI, −), total cross-sectional volume (mm^3^), cortical volume (mm^3^), marrow volume (mm^3^), cortical thickness (mm) and bone mineral density (BMD, mgHA/cm^3^) were analyzed.

### 4.4. Histomorphometry

Tibias were embedded undecalcified in methyl methacrylate (MMA). Five µm thick methyl methacrylate sections were cut using a Leica RM2255 microtome (Leica Biosystems, Nussloch, Germany) and stained with toluidine blue to quantify static measurements such as bone volume fraction (BV/TV, %), trabecular thickness (Tb.Th, μm), trabecular separation (Tb.Sp, μm), trabecular number (Tb.N,/mm), osteoblast surface per bone surface (Ob.S/BS, %), osteoclast surface per bone surface (Oc.S/BS, %) and eroded surface per bone surface (ES/BS, %). All histomorphometric analyses were carried out using the OsteoMeasure system (OsteoMetric Inc., Atlanta, GA, USA), following the standardized nomenclature [48].

### 4.5. qPCR Analysis

RNA was extracted from right femurs using TRIzol (Invitrogen, Waltham, MA, USA) and purified with the RNeasy Mini kit (Qiagen, Germantown, MD, USA) according to the manufacturers’ instructions. One μg of RNA was reverse-transcribed into cDNA by a SuperScript VILO cDNA synthesis kit (Invitrogen, Carlsbad, CA, USA). The qPCR was performed using Luna Universal qPCR master mix (New England Biolabs, Ipswich, MA, USA) at 57 °C for 39 cycles on a CFX96™ Optics Module (Bio-Rad, Hercules, CA, USA). The relative mRNA concentrations were normalized to *Gapdh*. A list of oligonucleotide primer sequences used for the qPCR analysis are showed in Supplementary Table S1.

### 4.6. Renal Histology

The left kidney was removed and fixed in 10% neutral formalin. The kidney was dehydrated and embedded in paraffin. Then, 5 μm thick sections were cut and stained with Masson’s trichrome. The renal interstitial fibrosis and tubular atrophy (IFTA) score was analyzed by computerized image analysis software (ImageJ software, version 1.38e, National Institutes of Health, Bethesda, MD, USA) using a 200× magnification, with 10 randomly selected fields per sample. A semi-quantitative scale was applied: 0, area of damage < 5%; 1, area of damage 5–10%; 2, area of damage 11–25%; 3, damage involving 26–50%; and 4, damage involving >50% of kidney section.

### 4.7. Serum Chemistry

The serum urea nitrogen, creatinine, phosphorus and calcium concentration were determined according to the manufacturer’s instructions (Standbio Laboratory, Boerne, TX, USA). Serum PTH levels were measured by an ELISA kit (Quidel, San Diego, CA, USA). Pro-inflammatory cytokine IL-23, IL1α, TNF-α, IFNγ, MCP-1, IL-12p70, IL-1β, IL-10, IL-6, IL-27, IL-17A, IFNβ and GM-CSF levels were determined using a multiplex beads-based assay (LEGENDplex^TM^) following the manufacturer’s instructions (BioLegend, San Diego, CA, USA.

### 4.8. Statistical Analysis

Data are expressed as mean ± standard error of mean. A one-way ANOVA, followed by a least significant difference (LSD) post hoc test, was used to analyze multiple comparisons. A two-way ANOVA was used to determine the interaction between *TβRI* overexpression and CKD. A *p*-value < 0.05 was considered statistically significant.

## Data Availability

All data are included in this article and its Supplementary Information File.

## Supplementary Materials

The following supporting information can be downloaded at: https://www.mdpi.com/article/10.3390/ijms26062704/s1.
