# Supplementary material for: Nephrectomy Induces Severe Bone Loss in Mice Expressing Constitutively Active TGFβ Receptor Type I
Source: Int J Mol Sci. 2025 Mar 17;26(6):2704. doi: 10.3390/ijms26062704 (PMC11943261; doi:10.3390/ijms26062704)
Supplement: Supplementary file 1 [file ijms-26-02704-s001.zip › ijms-3424528-supplementary.pdf]

Supplementary Table S1. List of oligonucleotide primers sequences used for qPCR analysis.

**Gene**	**Forward sequences (5’ to 3’)**	**Reverse sequences (5’ to 3’)**
Runx2	AGTCCCAACTTCCTGTGCTCC	CGGTAACCACAGTCCCATCTG
Sp7	CCCTTCTCAAGCACCAATGG	AAGGGTGGGTAGTCATTTGCATA
Tnfsf11	CAAGCTCCGAGCTGGTGAAG	CCTGAACTTTGAAAGCCCCA
Tnfrsf11b	AAGAGCAAACCTTCCAGCTGC	CACGCTGCTTTCACAGAGGTC
Acp5	GATCCCTCTGTGCGACATCA	CCAGGGAGTCCTCAGATCCA
Gapdh	TGCACCACCAACTGCTTAG	GGATGCAGGGATGATGTTC
